# Systems of care for cardiac arrest patients: Where are we going for tomorrow?

**DOI:** 10.1016/j.resplu.2024.100733

**Published:** 2024-07-31

**Authors:** Kasper G. Lauridsen, Shir Lynn Lim

**Affiliations:** Research Center for Emergency Medicine, Aarhus University Hospital, Denmark; Department of Anesthesiology and Critical Care, Randers Regional Hospital, Denmark; Department of Clinical Medicine, Aarhus University, Denmark; Department of Cardiology, National University Heart Centre, Singapore; Department of Medicine, National University of Singapore, Singapore; Pre-hospital and Emergency Research Centre, Duke-NUS Medical School, Singapore

**Keywords:** Basic Life Support, Advanced Life Support, Systems of Care

High-functioning systems of care are essential to improve survival outcomes for patients suffering from cardiac arrest.^1^ Building a high-functioning system of care, which covers the entire chain of survival, is complex. For out-of-hospital cardiac arrest (OHCA), this requires improving the community response (training of bystanders, dispatching of bystanders, public access defibrillation), enhancing Emergency Medical Services (EMS) (fast ambulance response times, well-trained paramedics), and advanced care such as extracorporeal life support.^2^ In the in-hospital setting, it spans from e.g. emergency medical teams for deteriorating patients, regular cardiopulmonary resuscitation (CPR) training of healthcare providers, and establishment of cardiac arrest teams.^3^ In both OHCA and in-hospital cardiac arrest (IHCA), quality post-arrest care is essential for good neurological recovery.

However, systems of care also need to be tailored to regional characteristics such as geography, sociocultural factors and available resources, further increasing complexity. Therefore, we have dedicated this special issue in Resuscitation Plus to the topic of Resuscitation Systems. The papers in this issue spanned across systems in high- and low-resource settings, OHCA and IHCA in pediatric and adult populations, pre- and post-arrest care (Table 1).

**Table 1:** Studies included.

**Overall Topics**	**Paper**
National pre-hospital systems of care	Kjærvoll HK et al. Description of the prehospital emergency healthcare system in Norway. https://doi.org/10.1016/j.resplu.2023.100509.
	Rensburg LV et al. A resuscitation systems analysis for South Africa: A narrative review. https://doi.org/10.1016/j.resplu.2024.100655
	Kabongo D et al. Evaluation of resuscitation systems in the Democratic Republic of Congo: A narrative review. https://doi.org/10.1016/j.resplu.2024.100656
	Thilakasiri K et al. “1990 Suwa Seriya” the national pre-hospital care ambulance service of Sri Lanka; a narrative review describing the EMS system with special emphasis on Out of Hospital Cardiac Arrest (OHCA) in Sri Lanka. https://doi.org/10.1016/j.resplu.2024.100649.
	Baig MNA et al. Pakistan's Emergency Medical Services (EMS) system & out-of-hospital-cardiac-arrest (OHCA): A narrative review of an EMS system of a low middle income country in context of OHCA. https://doi.org/10.1016/j.resplu.2024.100627.
	Okada Y et al. Novel and innovative resuscitation systems in Japan. https://doi.org/10.1016/j.resplu.2023.100541.
Specific system challenges to out-of-hospital cardiac arrest and international initiatives	Crause S et al. The barriers and facilitators to initiation of telephone-assisted bystander cardiopulmonary resuscitation for patients experiencing out-of-hospital cardiac arrest in a private emergency dispatch centre in South Africa. https://doi.org/10.1016/j.resplu.2023.100543.
	Gaisendrees C et al. Extracorporeal cardiopulmonary resuscitation for in- and out-of-hospital cardiac arrest: The race against time. https://doi.org/10.1016/j.resplu.2024.100613
	Zégre-Hensey JK et al. Challenges & barriers for real-time integration of drones in emergency cardiac care: Lessons from the United States, Sweden, & Canada. https://doi.org/10.1016/j.resplu.2024.100554
	Thies K et al. The European Trauma Course: Transforming systems through training. https://doi.org/10.1016/j.resplu.2024.100599
	Horriar L et al. Improving survival after cardiac arrest in Europe: The synergetic effect of rescue chain strategies. https://doi.org/10.1016/j.resplu.2023.100533
In-hospital cardiac arrest for adults and paediatrics	Haegdorens F et al. The third Medical Emergency Teams – Hospital outcomes in a day (METHOD3) study: The application of quality metrics for rapid response systems around the world. https://doi.org/10.1016/j.resplu.2023.100502
	Djärv T. Ten years of incident reports on in-hospital cardiac arrest – Are they useful for improvements? https://doi.org/10.1016/j.resplu.2023.100525
	Piscator et al. To withhold resuscitation – The Swedish system’s rules and challenges. https://doi.org/10.1016/j.resplu.2023.100501
	Mehta S et al. Implementation of a critical care outreach team in a children’s hospital. https://doi.org/10.1016/j.resplu.2024.100626
	Pedersen BBB et al. Organization and training for pediatric cardiac arrest in Danish hospitals: A nationwide cross-sectional study. https://doi.org/10.1016/j.resplu.2024.100555
Post-resuscitation care	Wagner MK et al. A multidisciplinary guideline-based approach to improving the sudden cardiac arrest care pathway: The Copenhagen framework. https://doi.org/10.1016/j.resplu.2023.100546
	Hunfeld M et al. Long-term multidisciplinary follow-up programs in pediatric cardiac arrest survivors. https://doi.org/10.1016/j.resplu.2024.100563

The first 6 papers describe systems of care for OHCA in unique settings with specific geographical, sociocultural and resource challenges: Norway, South Africa, the Democratic Republic of Congo, Sri Lanka, Pakistan, and Japan. The papers describe the current systems, their development, some of the existing challenges and future perspectives. Notably, description of systems of care in low-resource settings are sparse. Therefore, we are pleased to report unique descriptions of the systems of care in various low- and middle-income settings including their current prioritizations to improve survival outcomes from OHCA.

The next 5 papers describe unique parts and perspectives on the systems of care for OHCA patients. Crause et al. report qualitative findings on barriers and facilitators to telephone assisted cardiopulmonary resuscitation, highlighting important focus points for the South African system. Gaisendrees et al. identified factors associated with favorable outcomes following extracorporeal cardiopulmonary resuscitation, whereas Zégre-Hensey JK et al. report a narrative review on the specific challenges and opportunities for drones to deliver automated external defibrillators for OHCA. Moreover, we provide two unique aspects of initiatives to improve outcomes for OHCA, namely international initiatives of creating a European trauma course and different European initatives to improve rates of bystander CPR such as e.g. the kids save lives initiative.

Following this, we have 5 papers describing different aspects of IHCA among adult and pediatric patients. Among systems to prevent cardiac arrest, we present an international survey study on the use of rapid response teams and multiple parameter track and trigger systems and a unique single-center initiative for critical care outreach in a children’s hospital to facilitate early advanced care and transfer of deteriorating patients. In contrast, Pedersen et al. describes how treatment of pediatric cardiac arrest is organized in a healthcare system largely without children’s hospitals and how training of healthcare professionals in pediatric resuscitation varies widely. Djärv reports how to learn from mistakes through systematic reporting and follow-up of incidents that has led to various quality improvements over the years. Following this, Piscator et al. describes the Swedish system for do-not-attempt cardiopulmonary resuscitation aiming to ensure that patients are not resuscitated against their will and resuscitation is not initiated in patients where it is not judged to benefit the patients.

Finally, we report on two unique descriptions of post-arrest care, follow-up, and survivorship. Wagner et al. describes the Copenhagen framework for treatment of cardiac arrest survivors. This includes e.g. immediate post-arrest care with diagnostics in a specialized center and a comprehensive focus on screening for cognitive impairment and psychological problems as well as post-discharge rehabilitation with emphasis of activities of daily living. For pediatric patients, Hunfeld et al. describe how a comprehensive and interdisciplinary long-term follow-up of paediatric cardiac arrest survivors and their families has been established in the Netherlands including continuous assessment of motor function and neuropsychological function over several years.

Overall, this special issue exemplifies how clinicians and administrators in various settings and countries are working continuously to improve the systems of care. The papers demonstrate strengths and weaknesses as well as ambitions for future improvements, which collectively may serve as inspiration for other EMS and hospitals globally. However, we still lack information on the comparative effectiveness and cost-effectiveness of different system initiatives, a crucial consideration when prioritizing initiatives in resource-constrained regions.

Another question arising is where the future of the systems will take us. It is evident that the complexity of high-quality systems of care is increasing with more focus on technology considering everything from systems to dispatch laypersons to drones, helicopters, and prehospital application of extracorporeal life support.^4^, ^5^, ^6^, ^7^ Future systems may incorporate an even greater degree of technology including e.g. artificial intelligence and wearable devices and sensors to improve early recognition of cardiac arrest both in- and outside of hospitals.^8^, ^9^ Although the future is unknown, we must ask ourselves what we as clinicians can learn from the numerous examples provided in this special issue on Resuscitation Systems to improve our own systems for tomorrow.

## Funding

None.

## CRediT authorship contribution statement

**Kasper G. Lauridsen:** Writing – review & editing, Writing – original draft, Methodology, Conceptualization. **Shir Lynn Lim:** Writing – review & editing, Methodology, Conceptualization.

## Declaration of competing interest

The authors declare the following financial interests/personal relationships which may be considered as potential competing interests: [Dr. Kasper G. Lauridsen is the Young ERC editor at Resuscitation Plus. Dr. Shir Lynn Lim is an Associate Editor for Resuscitation and editorial board member at Resuscitation Plus. Dr. Lauridsen and Dr. Lim served as guest editors for the special issue on Resuscitation Systems at Resuscitation Plus.].
